# ABC of topical therapy in inflammatory bowel disease

**DOI:** 10.1002/jgh3.12323

**Published:** 2020-04-26

**Authors:** Thomas M Goodsall, Samuel P Costello, Robert V Bryant

**Affiliations:** ^1^ IBD Service, Department of Gastroenterology The Queen Elizabeth Hospital Adelaide South Australia Australia; ^2^ Faculty of Health Sciences, School of Medicine University of Adelaide Adelaide South Australia Australia

Refractory proctitis remains one of the most difficult conundrums in the management of inflammatory bowel disease (IBD). Although proctitis is more common in ulcerative colitis (UC), which affects the rectum and extends proximally in continuity, a proportion of patients with Crohn's disease (CD) also have disease that largely affects the rectosigmoid colon. Proctitis is often associated with debilitating symptoms of frequency, rectal bleeding, and incontinence despite limited disease extent.^1^ Topical therapies are efficacious in ulcerative proctitis and distal CD and have the advantage of single daily dosing, rapid onset of effect, and diminished systemic drug exposure and toxicity.^2^, ^3^ In this issue of *JGH Open*, Fehily *et al*. report their experience with a simple and accessible topical tacrolimus preparation in patients with UC and CD induced refractory proctitis, demonstrating both endoscopic and clinical response in two‐thirds of the cohort.

Despite the wealth of evidence, topical therapies are underused in IBD clinical practice.^1^ Although patient reluctance and tolerance play a role, it is also likely that clinician knowledge of rectal therapies is a contributory factor. A working knowledge of the ABCs of rectal therapy is an essential part of the IBD clinician's toolbox.

A. *5‐Aminosalicylates (5‐ASAs)* are first‐line therapies for UC. The use of combination oral and rectal 5‐ASA is superior to that of oral 5‐ASA alone for the induction and maintenance of remission.^3^ In proctitis (up to 18 cm from anal verge) or proctosigmoiditis (up to 50 cm from anal verge), topical therapies alone may be first‐line therapy.^2^, ^3^, ^4^ Symptomatic improvement is expected within 5–7 days. Topical 5‐ASAs are available as suppositories, foams, and enemas; choice is dictated by disease extent and patient tolerance. Given the multiple different preparations and applicator methods available, clinicians need to be armed with a practical knowledge of preparations in order to tailor therapy. In general, suppositories are well tolerated and best for proctitis. While enemas may provide more proximal drug delivery, issues with enema retention are common, in which setting a foam preparation may be trialed.^3^, ^4^ There is no benefit to dosing of >1 g per day for rectal 5‐ASA therapy. Adherence is far more important and may be improved by reducing dose frequency to three times a week, shown to maintain clinical and endoscopic remission in distal UC.^4^ Adverse events associated with topical 5‐ASAs are rare and comparable to placebo in most trials.^2^, ^3^, ^4^

*Acetarsol* is an organic arsenic compound that has been trialed as topical treatment for UC although it does not have regulatory approval from the Food and Drug Administration United States of America (FDA) or Therapeutic Goods Association Australia (TGA). Two recent retrospective trials showed positive results in refractory patients with proctitis. The first used acetarsol 250 mg suppositories twice daily (BD) in 39 patients and reported 68% clinical response and 82% endoscopic response (in a subgroup undergoing endoscopy) with only one reported adverse event of drug rash.^5^ The second used suppositories in 28 patients and reported a 68% clinical response although 21% discontinued acetarsol due to adverse events.^6^ Acetartsol is currently in the investigative space and further multicenter, prospective trials are required before its use can be recommended.

B. *Budesonide* is a second‐generation glucocorticoid with 90% first pass metabolism and low systemic bioavailability. Budesonide has been shown to be significantly more effective than placebo for inducing remission in UC in meta‐analysis data, with typical dosing of 2–4 g via liquid or foam enemas daily over 4 weeks.^2^ Despite having low bioavailability, rectal budesonide has been associated with decreased serum cortisol in 17.2% of patients and adrenal insufficiency in 3.7%, which may relate to rectal absorption bypassing the portal circulation.^7^ There are no trials of topical budesonide as long‐term maintenance for colitis.

*Beclomethasone dipropionate* is another second‐generation corticosteroid available as a 3 mg enema. A meta‐analysis demonstrated superiority of 5‐ASA over topical corticosteroids for induction of remission [relative risk (RR) 0.74, 95% confidence interval 0.61–0.90], which has rendered budesonide and beclomethasone second‐line topical therapies after failure or intolerance of 5‐ASA.^2^, ^3^, ^4^

C. *Corticosteroids* such as prednisolone (5 mg suppository or 20 mg/100 mL enema), betamethasone (5 mg/100 mL enema) and hydrocortisone (10% 21.1 g foam enema) are widely available and continue to have a role in topical therapy for induction of remission in UC. However, there is no evidence to support use of corticosteroids for maintenance of remission. Although hydrocortisone enemas may be comparable in efficacy to budesonide enemas, hydrocortisone and prednisolone are associated with a greater risk of suppression of the adrenocortical axis and systemic side effects.

*Calcineurin inhibitors* including tacrolimus and cyclosporine have been used systemically and topically in the management of IBD.^8^ *Topical tacrolimus* is thought to act locally as mucosal levels at biopsy may be high despite undetectable systemic levels.^9^

An Australian trial compared tacrolimus 1.5 mg rectal ointment BD with placebo and was closed after a planned interim analysis found clinical response in 73% of 11 patients receiving tacrolimus and 10% of 10 patients receiving placebo.^9^ In this issue, Fehily *et al*. report on the efficacy of a topical tacrolimus in tap water preparation in 17 IBD patients with refractory proctitis (12 UC and 5 CD). Administration of 1–3 g topical tacrolimus daily for a week and 3× per week thereafter for a median of 20 weeks achieved endoscopic response or remission in approximately two‐thirds of patients. Strikingly, the majority of these patients were refractory to immunomodulator and/or biologic therapy and rectal stricture resolution was noted in a subgroup of four patients. No significant adverse events were reported, which concurs with the low rate of adverse reactions to topical tacrolimus throughout the literature.^8^, ^9^

*Curcumin* via enema has been trialed in a small pilot randomised controlled trial (RCT) involving 45 patients with mild to moderate proctosigmoid UC (Mayo score 3–9) who were randomized to curcumin enema or placebo in addition to oral 5‐ASA 3.2 g/day.^10^ The primary outcome of decrease of three or more points on the Mayo score was achieved in 56.5% of the active group and 36.4% of the placebo group (*P* = 0.18) but was significant on an intention to treat analysis (92.9 *vs* 50%, respectively, *P* = 0.01). Curcumin is thought to act through inhibition of nuclear factor kappa B induced cytokine release and further trials are warranted.

In summary, topical therapies are a valuable part of the therapeutic armamentarium for distal IBD, both for UC and CD, and further randomized controlled trials are needed to clarify the role of investigative rectal treatment options. A practical working knowledge of the ABCs of topical therapy in IBD is required to facilitate treatment decisions, to improve patient compliance, and ultimately to improve patient outcomes.
